# The MEK5/ERK5 Pathway in Health and Disease

**DOI:** 10.3390/ijms22147594

**Published:** 2021-07-15

**Authors:** Rupesh Paudel, Lorenza Fusi, Marc Schmidt

**Affiliations:** Department of Dermatology, Venereology and Allergology, University Hospital Würzburg, 97080 Würzburg, Germany; Paudel_R@ukw.de (R.P.); Fusi_L@ukw.de (L.F.)

**Keywords:** atherosclerosis, bone, cartilage, endothelium, extracellular-regulated kinase 5, Krüppel-like factor, mechanotransduction, mitogen-activated protein kinase, stress signaling, tumor

## Abstract

The MEK5/ERK5 mitogen-activated protein kinases (MAPK) cascade is a unique signaling module activated by both mitogens and stress stimuli, including cytokines, fluid shear stress, high osmolarity, and oxidative stress. Physiologically, it is mainly known as a mechanoreceptive pathway in the endothelium, where it transduces the various vasoprotective effects of laminar blood flow. However, it also maintains integrity in other tissues exposed to mechanical stress, including bone, cartilage, and muscle, where it exerts a key function as a survival and differentiation pathway. Beyond its diverse physiological roles, the MEK5/ERK5 pathway has also been implicated in various diseases, including cancer, where it has recently emerged as a major escape route, sustaining tumor cell survival and proliferation under drug stress. In addition, MEK5/ERK5 dysfunction may foster cardiovascular diseases such as atherosclerosis. Here, we highlight the importance of the MEK5/ERK5 pathway in health and disease, focusing on its role as a protective cascade in mechanical stress-exposed healthy tissues and its function as a therapy resistance pathway in cancers. We discuss the perspective of targeting this cascade for cancer treatment and weigh its chances and potential risks when considering its emerging role as a protective stress response pathway.

## 1. Introduction

Extracellular signal-related kinase (ERK) 5 belongs to the family of conventional mitogen-activated protein kinases (MAPK) [1]. Besides ERK5, this family of serine/threonine kinases comprises the classical growth factor-activated members ERK1 and ERK2 [2], and the stress-activated protein kinases (SAPK) p38 (p38α-δ) and c-Jun N-terminal kinase (JNK1-3) [3,4]. MAPK pathways are activated by a wide variety of stimuli, including mitogens, cytokines, stress, and UV radiation [1]. They all comprise a three-tiered signaling module with MAPK kinase kinases (MAP3Ks or MEKKs) at the top of the cascade. MEKKs phosphorylate downstream MAPK kinases (MAP2Ks or MEKs), which in turn phosphorylate and activate the ultimate effector MAPKs: ERK1/2, ERK5, p38, and JNK. The activated MAPKs shuttle into the nucleus where they phosphorylate diverse substrates, including transcription factors, co-regulators and chromatin proteins. These activating or inactivating phosphorylation events regulate cell survival, proliferation, migration, differentiation, and angiogenesis, as well as apoptotic signaling (reviewed in [1,2,3]).

ERK5, encoded by the *MAPK7* gene, is also referred to as big MAP kinase 1 (BMK1) [5] due to its large and unique C-terminus. Unlike the primarily growth factor-activated ERK1/2 and the mainly stress-activated SAPKs, ERK5 can be activated by both mitogenic stimuli, such as EGF and FCS [6,7], and stress factors, including high osmolarity and fluid shear stress [8,9]. Since its discovery in 1995 [5,10], efforts have been made to explore its physiological role and relevance in disease. Earlier reviews [4,11] published almost a decade ago provided insights into the structural and functional role of ERK5, whereas recent reviews have focused on its contribution to oncogenesis and the perspective of targeting ERK5 in cancer [12,13,14,15,16]. Here, we briefly discuss the current view on the regulation of ERK5 and summarize our knowledge of its role in health and disease. In particular, we will focus on its function as a mechanoreceptive and stress-responsive pathway in the endothelium and other stress-exposed tissues. Additionally, we summarize its emerging role as a drug resistance pathway in various cancers.

## 2. The MEK5/ERK5 Pathway

### 2.1. ERK5: Structure and Regulation

Structurally, ERK5 differs from its closest relative, ERK2, and other MAPKs by the presence of a unique C-terminal extension. This extended C-terminus accounts for the extraordinary size of ERK5, which is approximately twice the molecular weight of other MAPKs and fulfills a regulatory function [1,4]. In the absence of an activating stimulus, it folds back on its kinase domain located at the N-terminus. This exposes a nuclear export signal that keeps ERK5 in a closed inactive conformation that is bound to a cytosolic anchor composed of the chaperone HSP90 and its co-chaperone CDC37 [17,18]. Phosphorylation at a TEY motif at threonine 219 and tyrosine 221 in the kinase activation loop of ERK5 via its upstream MAP2K MEK5 (MAP2K5) unleashes ERK5 kinase activity. This interaction between both kinases is facilitated via a specific Phox and Bem1 (PB1) protein dimerization/oligomerization domain present in MEK5, which is absent in other MEKs, and confers specificity to the interaction [19]. As a result of MEK5-dependent TEY phosphorylation, ERK5 auto-phosphorylates several sites at its C-terminus (Table 1), which is visible in immunoblots by the appearance of a distinct slower migrating band [20]. The C-terminal phosphorylation of ERK5 then triggers a conformational change, resulting in the dissociation of HSP90 and exposure of a hidden C-terminal nuclear localization signal facilitating nuclear entry (reviewed in [21]). Inside the nucleus, ERK5 subsequently stimulates transcription both via phosphorylation of transcription factors such as MEF2C [6], and via an enigmatic direct mechanism, which involves transcriptional activation through a C-terminal transactivation domain (TAD) [22].

Intriguingly, a recent mutagenesis study highlights a particular gatekeeper role of T733 phosphorylation in this C-terminal TAD. Expression of a phosphomimetic ERK5 mutant of T733 alone was able to stimulate activation of an MEF2-dependent reporter, even in the absence of additional MEK5 activation and permitted nuclear localization of ERK5 under basal conditions [27]. Phosphorylation at this site commonly results from ERK5 autophosphorylation as a consequence of MEK5-induced TEY phosphorylation [20] coupling MEK5-driven ERK5 kinase activity with its transactivation capacity. However, several reports support the view that the kinase and transactivation function of ERK5 can also be uncoupled under certain circumstances. For instance, CDC37 overexpression was able to trigger nuclear localization and transactivation of an ERK5-responsive AP-1 reporter independently of MEK5 and ERK5 activity [17]. Moreover, some studies have reported alternative phosphorylation of T733 and other C-terminal sites in the absence of MEK5 and ERK5 kinase activity (Table 1). For example, a mitotic arrest induced by the spindle poison nocodazole was able to induce the phosphorylation of ERK5 at T733 in mouse embryonic fibroblasts (MEFs) with *Mek5* deficiency [25,26]. Similarly, nocodazole treatment triggered T733 phosphorylation in *Erk5*^−/−^ MEFs supplemented with kinase-dead ERK5 and was capable of inducing T733 phosphorylation in an ectopically expressed TEY mutant of ERK5 [25,26]. Both studies showed that nocodazole-induced T733 phosphorylation was sensitive to CDK1 inhibition, but none of the groups were able to show the direct CDK1-dependent phosphorylation of ERK5 T733 [25,26]. In another report, alternative T733 phosphorylation of ERK5 was induced upon expression of oncogenic RAS V12. Since T733 phosphorylation was sensitive to ERK1/2 inhibition using the MEK inhibitor U0126 and occurred in the absence of TEY phosphorylation or obvious ERK5 kinase activity, the authors concluded that T733 phosphorylation of ERK5 resulted from ERK2 activation [23]. Indeed, they were able to demonstrate co-immunoprecipitation of ERK2 and ERK5 but failed to provide direct evidence for ERK2-mediated T733 phosphorylation [23]. TEY-independent T733 phosphorylation of ERK5 has also recently been found to occur in colorectal cancer cells overexpressing CDK5 [24]. Here, the authors validated T733 phosphorylation of immunoprecipitated ERK5 in kinase assays with recombinant CDK5. Surprisingly, however, they observed a sensitivity of CDK5-mediated T733 phosphorylation to the MEK5 inhibitor BIX02189 [24], raising questions regarding the exact mechanism and/or specificity of the employed inhibitor.

### 2.2. ERK5 in Proliferation

Based on the high similarity between the kinase domains of ERK2 and ERK5 and their similar activation by growth factors, initial in vitro work established ERK5 as an additional MAPK that controlled proliferation. In this context, ERK5 was identified as an effector kinase of oncogenic HRAS that triggered phosphorylation of c-MYC [28]. ERK5 was also able to promote growth factor-dependent proliferation via phosphorylation of the transcription factor MEF2C and the subsequent induction of immediate early genes, such as the AP-1 transcription factor *c-JUN* [6]. Additionally, ERK5 was proposed to control G1-S cell cycle progression by the regulation of serum and glucocorticoid kinase (SGK1) [29], cyclin D1 [30], or promyelocytic leukemia protein- (PML-) dependent suppression of the cyclin-dependent kinase inhibitor p21 [31]. Hence, ERK5 may regulate proliferation through multiple mechanisms (reviewed in [32]).

While these early studies fueled interest in targeting ERK5 for cancer therapy, genetic studies performed in the early years of the 21st century revealed an essential role of ERK5 and MEK5 in cardiovascular development. These investigations demonstrated a key role of ERK5 in both embryonic heart development, angiogenesis, and the maintenance of endothelial function and survival in adult mice (discussed below) but failed to provide conclusive evidence for a non-redundant role in proliferation [33,34,35]. Consistently, newer data using specific kinase inhibitors for the MEK5/ERK5 pathway, as well as siRNA depletion experiments, demonstrated that tumor cells with oncogenic KRAS or BRAF mutations or *ERK5* amplification were not addicted to ERK5 activity for proliferation [36]. In agreement, *ERK5* knockdown in NRAS-mutant melanoma cells with increased basal ERK5 activity only transiently impaired the G1-S progression but did not affect long-term proliferation [37]. Considering its dual function as a stress- and mitogen-activated pathway, the MEK5/ERK5 cascade therefore might rather be viewed as a non-essential “escape pathway” that offers tumor cells a proliferation advantage under drug- or oncogene-induced stress conditions.

### 2.3. Physiological Functions of the MEK5/ERK5 Pathway Revealed by Knockout Studies

While the above-cited examples of non-canonical ERK5 phosphorylation indicate the existence of a context-specific ERK5 regulation independent of MEK5, there is overwhelming genetic evidence to suggest that most of its physiological functions require MEK5 activity. For instance, targeted knockout mice for *Erk5* or *Mek5* had strikingly similar phenotypes and were characterized by cardiovascular defects in heart development, vessel maturation, angiogenesis, and endothelial integrity, leading to embryonic lethality at around stage E10 [34,35]. Analogous effects were observed in mice, in which *Erk5* was selectively knocked out in endothelial cells [33]. Cardiomyocyte-specific *Erk5* knockout mice, however, were largely normal [33]. Thus, the cardiac phenotype primarily appears to evolve as a consequence of endothelial dysfunction. Of note, the conditional deletion of *Erk5* in adult mice resulted in lethality within 2–4 weeks due to vascular leakage associated with massive endothelial apoptosis [33], suggesting that ERK5 function is also required to sustain the endothelial integrity of adult mice. Remarkably, the time frame of lethality of both the endothelial-specific *Erk5* knockout mice and the global *Mek5* and *Erk5* knockout mice coincides with the establishment of a fully functional circulation and the onset of embryonic blood flow [38]. This indicates an intimate relationship between MEK5/ERK5 activation and the induction of major vasoprotective effects of laminar shear stress, as discussed below.

## 3. ERK5 in Mechanical Stress-Exposed Tissues and MAPK Inhibitor-Resistant Cancers

For the sake of brevity and coherence, we limit our review to a discussion of both the MEK5/ERK5 pathway in tissues particularly exposed to mechanical stress and therapeutic options for drug-resistant cancers. We refer the interested reader to excellent reviews published elsewhere regarding its role in neuronal survival [39,40], and in general aspects of cancer biology [12,15].

### 3.1. Endothelium

There is ample evidence showing that ERK5 plays a crucial role in maintaining homeostasis of the cardiovascular system, especially in the vascular endothelium. The vascular endothelium is an interface composed of a monolayer of endothelial cells (ECs) between the blood and the blood vessel wall. The flow of blood through the healthy vessels exerts a steady laminar shear stress (LSS) on the endothelial lining. This steady LSS plays an important role for endothelial integrity by enhancing the apoptosis resistance of ECs and inducing a quiescent state characterized by decreased endothelial permeability, migration, angiogenesis, and steady-state inflammation. Additionally, it leads to the increased expression of anti-thrombotic genes, and to the suppression of vascular smooth muscle cell proliferation (reviewed in [41,42]).

**Figure 1:**
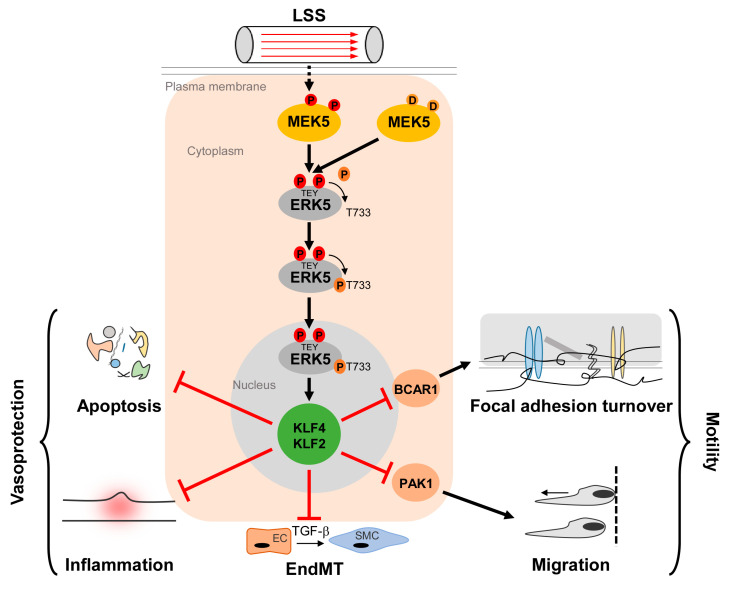
Activation and function of the MEK5/ERK5 pathway in vascular endothelium. Laminar shear stress (LSS) induced by the steady blood flow through the vasculature results in phosphorylation of MEK5 in ECs, which subsequently phosphorylates ERK5 at a TEY-motif at threonine 219 and tyrosine 221 [9] (highlighted in red color). Subsequently, ERK5 autophosphorylates its C-terminus at multiple sites (including T733, shown as representative residue in dark orange) [20], leading to a change in conformation and nuclear localization of ERK5. Within the nucleus, ERK5 induces protective gene expression via transcriptional induction of the Krüppel-like factor 2 and 4 (KLF2/KLF4) transcription factors, thereby inhibiting apoptosis, inflammation, focal adhesion turnover, migration, and endothelial to mesenchymal transition (EndMT) [43,50,51,52,53]. Even though KLF2 and KLF4 can act functionally redundant in an overexpression situation, some functions may dominantly be controlled by only one of the two KLFs. For example, KLF2 induction alone is responsible for PAK1 repression, which contributes to the anti-migratory effect of ERK5 activation [51]. By contrast, BCAR1 suppression, which has been implicated in the inhibition of focal adhesion turnover [50] requires both KLF2 and KLF4 [51].

The MEK5/ERK5 pathway is long known to be activated by LSS and to promote apoptosis resistance of ECs [9,43,44]. However, its acceptance as a key mediator of protective shear stress responses steeply increased through the discovery that the transcription factor Krüppel-like factor 2 (KLF2, also known as lung Krüppel-like factor (LKLF)) is a crucial ERK5 effector that is transcriptionally downregulated in *Erk5*^−/−^ fibroblasts [45]. *KLF2* is a well-established flow response gene induced by protective laminar flow but not by disturbed flow patterns [46,47] that prevail in arterial areas susceptible to inflammation and atherosclerotic cardiovascular disease [41]. KLF2 expression is restricted to lateral parts of the arteries that are protected from atherosclerotic plaque formation but is absent at sites prone to plaque deposition [46]. Furthermore, its overexpression results in various transcriptional changes typical for atheroprotective flow patterns [47,48]. *KLF2* carries a binding site for MEF2 in its proximal promoter and *KLF2* induction by atheroprotective flow occurred in an MEF2- and MEK5-dependent manner [47]. Accordingly, the constitutive ERK5 activation through the expression of an active MEK5 mutant (MEK5D) resulted in a gene expression pattern similar to that induced by vasoprotective flow or KLF2 overexpression [43,49]. It further induced a phenotype characterized by a decreased susceptibility to inflammatory, apoptotic, and migratory cues [43,50] (Figure 1).

**Figure 2:**
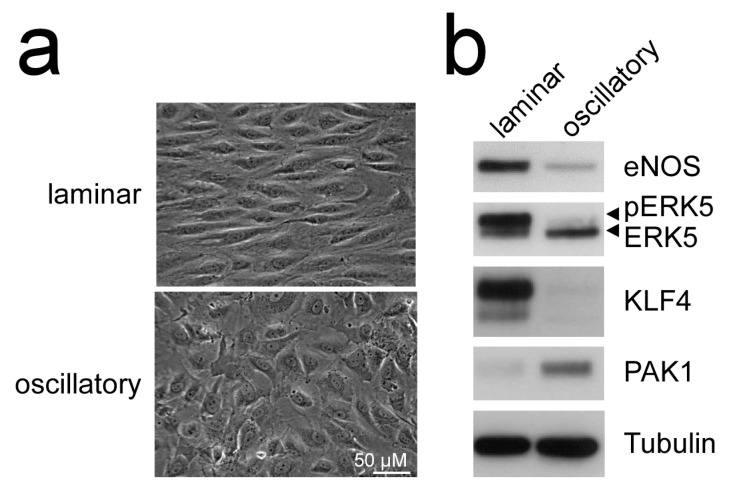
Laminar flow but not oscillatory flow activates the MEK5-ERK5-KLF axis: (**a**), phase contrast photographs of human umbilical vein endothelial cells (HUVEC) subjected to laminar shear stress (20 dyne/cm^2^) or oscillatory flow (2 Hz) for 120 h, as described [51,52]; (**b**), immunoblots of total cell lysates from the differently treated cells. Only laminar flow is able to induce ERK5 activation, as evident by appearance of a slower migrating band corresponding to C-terminally phosphorylated ERK5 [20], induction of KLF4 [43,49], the KLF2-target eNOS [53], and suppression of PAK1 [51] protein. Tubulin expression is shown as loading control.

Additionally, MEK5D expression and laminar flow, but not disturbed flow, increased transcription of another Krüppel-like factor, KLF4 [43,49,51] (Figure 2), where its overexpression could partially reproduce the anti-inflammatory, anti-apoptotic, and migration-inhibiting effects of active ERK5 in primary human endothelial cells [43]. Similar to KLF2, KLF4 induction is supposed to rely on MEF2 activation, as MEF2C was found to bind to a distal enhancer in the *KLF4* gene [54]. Arterial KLF4 expression is also restricted to areas exposed to laminar flow but absent at sites of disturbed flow [55]. Of note, both KLFs may act partially redundant, as indicated by genetic complementation experiments and the co-regulation of target genes in overexpression studies. For instance, forced KLF2 or KLF4 expression could both inhibit endothelial proinflammatory activation [43,53,56] and induce antithrombotic gene expression or expression of the vasodilatory gene eNOS [43,57].

Conversely, the inducible endothelial-specific deletion of *Klf2* and/or *Klf4* in mice revealed that a single allele of *Klf2* and *Klf4* was sufficient for survival [58]. The endothelial-specific loss of all *Klf2* and *Klf4* alleles severely compromised the vessel integrity and led to vascular leakage, coagulation failures, and acute death from myocardial infarction, heart failure, and stroke [58]. This is consistent with a partial redundancy of KLF2 and KLF4 in early development. Similar to the endothelial-specific knockouts, the combined germline deletion of *Klf2* and *Klf4* resulted in a more severe phenotype than the deletion of any of the single *Klf* genes, and *Klf2/4*^−/−^ embryos died in utero with signs of hemorrhage at the same stage as the knockouts for *Mek5* or *Erk5* [59]. The described phenotype of the *Klf2/4* double knockouts was reminiscent of the phenotype produced by the conditional *Erk5* deletion [33] and partially overlapped with the phenotype of mice with the endothelial-specific loss of *Mef2a/c/d* [60].

Consistent with a proposed role of ERK5, KLF2, and KLF4 in atheroprotection, various mouse models of atherosclerosis further established a key role of the ERK5/KLF2/KLF4 cascade as a flow-regulated signaling module that safeguards from atherosclerosis [61,62,63]. For instance, both hemizygous *Klf2* germline deletion or endothelial-specific *Klf4* loss sensitized atheroprone apolipoprotein E–deficient (*ApoE*^−/−^) mice to high fat diet-induced atherosclerosis [61,64]. Conversely, expression of an EC-specific *Klf4* transgene protected *ApoE*^−/−^ mice from high fat diet-induced atherosclerosis [64]. Similarly, the conditional endothelial loss of *Erk5* increased high fat-diet induced atherosclerosis in susceptible mice [63]. This phenotype was associated with increased expression of inflammatory endothelial surface proteins, such as VCAM1, which promotes leukocyte adhesion [63], corroborating earlier observed immunosuppressive effects of ERK5 activation in human primary ECs [43]. Interestingly, Moonen et al. found that neointimal hyperplasia characterizing initial stages of human atherosclerosis was associated with an increased transdifferentiation of ECs into highly motile and fibroproliferative vascular smooth muscle cells [52]. This transdifferentiation process, termed endothelial to mesenchymal transition (EndMT), was shown to correlate with increased expression of mesenchymal markers and calcification, and was induced in EC cultures by exposure to disturbed flow in the presence of TGFβ [52]. The laminar flow or endothelial MEK5D expression, by contrast, suppressed EndMT and calcification and were associated with the ERK5-dependent induction of *KLF2* and *KLF4* expression [52]. Thus, the different flow patterns apparently directly influence the development of fibroproliferative vascular disease by modulating the ERK5-dependent EC differentiation.

**Figure 3:**
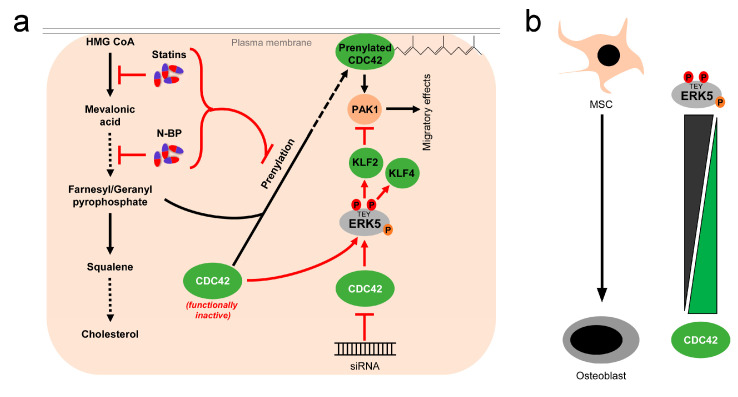
Activation of ERK5 by drugs inhibiting the mevalonate pathway and correlation between CDC42 function and ERK5 activity: (**a**), 3-hydroxy-3-methylglutaryl-coenzym-A-reduktase inhibitors (statins), a group of cholesterol-lowering drugs, and N-bisphosphonates (N-BP), used for osteoporosis treatment, activate ERK5 via inhibition of the mevalonate biosynthesis pathway. By inhibition at different stages of the mevalonate cascade, both drugs interfere with prenylation and subsequent membrane localization of small GTPases such as CDC42, thus stalling them in a functionally inactive stage [71]. Functional inactivation of CDC42 leads to elevated ERK5 activity, which can be also mimicked by siRNA-mediated knockdown of *CDC42* [73,74]. Activated ERK5 also inhibits PAK1 expression, thereby interfering with downstream effects of CDC42 [51]; (**b**), inverse correlation of ERK5 and CDC42 activity in mesenchymal stem cells (MSC) and differentiated osteoblasts. While ERK5 activity is high in MSCs, it drops during osteogenic differentiation [73,75]. Conversely, CDC42 activity is low in MSCs and rises during the differentiation process [73,76].

While these reports overall suggest the existence of a linear flow-activated MEK5/ERK5/MEF2/KLF2/KLF4 pathway in ECs sustaining vascular integrity (Figure 1), the exact upstream events triggering activation of the pathway in ECs are still poorly understood. Genetic evidence implies the MAP3K MEKK3 as the most likely candidate for the kinase activating MEK5 in the endothelium [65,66]. However, MEKK2 could also bind to MEK5 via its PB1 domain, and was required for sorbitol-induced ERK5 activation in other cells [67]. Even less information is available regarding the upstream activation of MEKK2/3. Interestingly, we, along with others, have shown that ERK5, as well as its downstream effectors KLF2 and KLF4, could also be activated by vasoprotective 3-hydroxy-3-methyl-glutaryl-coenzyme A (HMG-Co-A) reductase inhibitors (“statins”) [43,49,68,69]. Statins are drugs frequently used for the treatment of hyperlipidemia, due to their cholesterol-lowering capacity. However, beyond their hypolipidemic actions they also have other beneficial effects on the vasculature. For instance, they act anti-inflammatory and can stabilize atherosclerotic plaques [70]. These pleiotropic effects of statins have been attributed to their interference with an early enzymatic step of the mevalonate biosynthesis pathway, which not only controls cholesterol synthesis but also the prenylation of small GTPases of the RhoA family [71], which critically regulate cytoskeletal functions and motility [72] (Figure 3a). Consequently, the statin-mediated inhibition of the mevalonate pathway hampers the proper functioning of small GTPases such as CDC42. Notably, we have recently observed that another class of mevalonate-inhibiting drugs, the nitrogen-containing (N-) bisphosphonates (N-BP), which are used for the treatment of osteoporosis, can also trigger ERK5 phosphorylation and *KLF2* and *KLF4* induction in various cells, including ECs and differentiated primary human osteoblasts [73]. These drugs interfere with a further downstream step of the mevalonate pathway, suggesting that statins and N-BPs may regulate ERK5 via a common mechanism (Figure 3a). Intriguingly, we were able to show that *CDC42* knockdown alone was sufficient to induce ERK5 phosphorylation and KLF2/4 induction in human primary ECs [73]. *CDC42* knockdown was also able to induce ERK5 activity in breast cancer cells, and CDC42 activation suppressed ERK5 phosphorylation in those cells [74]. Additionally, we provided evidence that forced ERK5 activation in endothelial cells results in the KLF2-dependent inhibition of P21-activated kinase 1 (PAK1) expression, an important promigratory effector of active RAC and CDC42, thereby inhibiting endothelial migration [51]. Thus, CDC42 and ERK5 signaling may interact at different levels (Figure 3a).

Genetic mouse models of cerebral cavernous malformations (CCM) further support the assumption of an inverse correlation between ERK5 and CDC42 activity. CCM is a severe vascular disease of capillaries in the brain which frequently leads to cerebral hemorrhage. Two different groups independently reported that familial CCM caused by the loss of function mutations in CCM genes resulted in an increased activation of the MEKK3/MEK5/ERK5/KLF2/KLF4 pathway and the development of CCM lesions that were ameliorated by the endothelial loss of *Mekk3*, *Klf2*, *or Klf4* [66,77]. Castro et al. subsequently reported that the postnatal endothelial deletion of *Cdc42* also led to increased MEKK3/MEK5/ERK5/KLF2/KLF4 signaling and CCM lesions, where its severity was reduced by the additional loss of *Klf4* [78]. These data suggest that CDC42 inhibition, induced by cytoskeletal changes, might partly account for the activation of the MEK5/ERK5 pathway.

### 3.2. Bone and Cartilage

Bone health critically depends on a balance between the opposing actions of bone-forming osteoblasts produced by differentiation of mesenchymal stem cells (MSCs) and bone-resorbing osteoclasts differentiating from the myeloid lineage [79]. Osteoporosis, a bone degenerative disease of elderly people, exemplarily illustrates the importance of bone homeostasis. Osteoporosis develops when anabolic activity is lower than catabolic activity in bone, which is frequently associated with a decreased osteoblast production and function [79]. Many studies imply that in analogy with the endothelium, mechanical forces importantly influence bone homeostasis. Mechanical stress on the bone positively correlates with bone mass [80], as evidenced by the increased bone resorption in demographical groups whose bones are not exposed to mechanical stress. Bed-ridden patients with limited mobility [81] and astronauts who are frequently subjected to microgravity [82] belong to such groups.

A currently popular concept proposes that the maintenance and restoration of bone tissue is strongly influenced by the shear stress generated in the lacunar–canalicular network by the flow of the interstitial fluid [83]. This view is reminiscent of the conditions in the vascular endothelium, in which ERK5 plays a crucial role in mediating the shear stress response. Indeed, fluid shear stress could also trigger ERK5 phosphorylation in murine MC3T3-E1 preosteoblasts [84]. This promoted osteoblast proliferation via an AP-1/cyclin D dependent pathway [84] and supported osteoblast survival via the inhibition of TNFα-induced apoptosis by AKT-dependent suppression of the proapoptotic transcription factor FoxO3 [85]. Additionally, ERK5 was proposed to mediate the differentiation of MC3T3-E1 osteoblasts by intermittent fluid shear stress, as evidenced by the suppression of flow-induced expression of several osteoblast differentiation markers, including alkaline phosphatase (ALP), osteopontin, and osteocalcin, in the presence of a pharmacological ERK5 inhibitor [86].

Our own studies further support the hypothesis that ERK5 may contribute to a sustained bone homeostasis. We provided first hints that ERK5 activation in ECs was not only capable of inducing expression of vasoprotective genes, but also significantly upregulated several genes relevant for calcium metabolism and bone regulation, such as the parathyroid hormone-like hormone (*PTHLH*) [43,73]. PTHLH is an important hormone involved in the regulation of calcium and bone homeostasis and was recently shown to promote bone matrix strength by regulating bone mineralization without affecting osteoclastogenesis [87]. In part, this may contribute to the clinical success of a synthetic PTHLH-derived peptide drug, abaloparatide, in the treatment of osteoporosis in postmenopausal women [88]. Interestingly, we observed that during osteoblast, the differentiation of human MSCs *PTHLH* expression strictly correlated with ERK5 activity that was high in undifferentiated MSC, but gradually lost during the differentiation into osteoblasts [73]. By contrast, CDC42 GTPase activity inversely correlated with ERK5 phosphorylation during osteogenic differentiation and was found to be low in MSCs but increased in differentiated osteoblasts [73] (Figure 3b). These findings are in agreement with earlier studies that reported a high basal level of ERK5 phosphorylation in adult multipotent progenitor cells from human bone marrow [75] and an increased CDC42 activity during osteogenic differentiation, which contributed to the osteoblast differentiation of human MSCs [76]. Of note, treatment with the N-BP zoledronate, a drug commonly used for osteoporosis treatment that, in part, mediates its effects via the functional inhibition of the mevalonate pathway and small GTPases (Figure 3a), restored ERK5 activity in differentiated human MSC-derived osteoblasts, and re-induced *PTHLH* expression in those cells via an ERK5- and KLF2-dependent mechanism [73]. Thus, besides their established function as inducers of osteoclast apoptosis, N-BPs may also exert their bone-sustaining effects by the ERK5 reactivation and subsequent stimulation of bone mineralization. Consistent with a key role of ERK5 in bone mineralization, ERK5 inhibition, by small hairpin-mediated depletion or pharmacological ERK5 inhibition using the small-molecule inhibitor XMD8-92, disturbed the proper timing and induction of mineralization genes such as *ALP* during osteogenic differentiation [73]. However, ERK5 activity was dispensable for osteogenic commitment per se, as the induction of *RUNX2*, a master regulator of this process [79], occurred normally when ERK5 was inhibited [73].

Recent genetic data further support a role of ERK5 in bone regulation. The conditional deletion of *Erk5* in mice by Nkx3.1:Cre, which was originally intended to delete *Erk5* specifically in the prostate, surprisingly resulted in skeletal disorganization and osteopenia, a condition of increased bone resorption [89]. In this case, the bone loss was proposed to result from increased osteoclastogenesis, suggesting that ERK5 may also inhibit osteoclast differentiation. Similarly, the inactivation of *Erk5* in mesenchymal cells of the limb bud and craniofacial mesenchyme by the paired-related homeobox 1- (Prx1-) Cre-mediated conditional deletion of *Erk5* hampered the skeletal development. This was partially attributed to a delayed or disturbed mineralization in metatarsal, metacarpal, and proximal phalanges [90], establishing a role of ERK5 in bone mineralization.

However, seemingly contradictory results have also emerged. The inhibition, rather than stimulation, of the MEK5/ERK5 pathway by the selective dual MEK5/ERK5 inhibitor BIX02189 was shown to accelerate mineralization and osteoblast differentiation via an osterix-dependent mechanism [91]. Moreover, macrophage colony-stimulating factor (M-CSF)-activated ERK5 activation was found to be crucial in the differentiation of 4B12 precursor cells into osteoclasts [92].

There is also limited evidence to suggest that ERK5 activity may contribute to chondrogenesis, i.e., the production of chondrocytes, the main cells of cartilage, which cover and shield the end of the bones at the joints. Chondrocytes play an important role in the embryogenic formation of both the axial skeleton (vertebrae and ribs) and the appendicular skeleton (limbs), which occurs by endochondral ossification, a process in which bone systematically replaces the growing cartilage in order to form the final skeleton [79]. Unlike in intramembranous ossification, which is responsible for the development of non-long bones, such as the bones of the skull and clavicle, osteoblast differentiation during endochondrial ossification does not occur directly by condensation of the mesenchymal progenitors. Instead, it requires an intermediate step in which mesenchymal progenitor cells first differentiate into pre-osteoplastic perichondrial cells and chondrocytes. Subsequently, chondrocyte hypertrophy in the primordium triggers the differentiation of perichondrial cells into mature osteoblasts [79]. Hence, in endochondral ossification, osteoblasts and chondrocytes share a common ancestry and their differentiation is tightly connected, raising the question of whether the MEK5/ERK5 pathway might influence chondrogenesis. Regarding this issue, ERK5 was previously found to downregulate the expression of *Col2a1* and *Sox9*, two well-established factors regulating chondrocyte differentiation in MSCs [93]. Conversely, the siRNA-mediated knockdown of *MEK5* and *ERK5* in MSC-derived human bone marrow-derived multipotent progenitor cells resulted in increased expression of SOX9 and COL2A1 and the induction of several cartilage-characteristic marker genes [75], suggesting that ERK5 may suppress chondrogenesis. The conditional loss of *Erk5* in mesenchymal cells in the above-mentioned Prx1-Cre system also resulted in increased SOX9 expression, and the loss of one allele of *Sox9* could rescue several skeletal defects in the mice [90]. However, careful phenotypic analysis of the mesenchymal ERK5-deficient mice revealed that chondrogenesis was delayed, rather than suppressed, as evident by the lack of chondrocyte hypertrophy and the exclusive expression of COL2A1, a marker of non-hypertrophic chondrocytes, by the metatarsal chondrocytes [90]. Interestingly, the specific conditional deletion of *Erk5* in the chondrocytes of postnatal mice also disturbed skeletogenesis, resulting in animals characterized by growth restriction, short limbs, and bone mass loss [94]. This phenotype was likewise attributed to impaired endochondral ossification and chondrocyte hypertrophy, as the mice showed decreased chondrocyte survival and proliferation in the hypoxic center of the proliferative layer [94]. Taken together, these reports imply a requirement for the proper timing and induction of chondrocyte hypertrophy rather than a general suppressive role of ERK5 in chondrogenesis. In line with this notion, the deletion of *Mef2c* in endochondrial cartilage impaired chondrocyte hypertrophy, whereas expression of a super activating MEF2C caused precocious chondrocyte hypertrophy, ossification of growth plates, and dwarfism [95].

With respect to the emerging evidence for a potential antagonism between ERK5 and CDC42 signaling, it is also worth mentioning that the genetic disruption of *Cdc42*, *Cdc42* knockdown, or inhibition of CDC42 activity significantly improved neovascularization and bone loss in an experimental mouse model of osteoarthritis [96]. CDC42 inhibition also restored numbers of MSCs, osteoprogenitors, osteoblasts, and osteoclasts, which normally drop in this mouse model [96]. Unfortunately, the authors did not analyze the impact of CDC42 inhibition on ERK5 activity. Nonetheless, it is tempting to speculate that ERK5 re-activation might, at least partially, account for the improved cartilage maintenance observed upon *Cdc42* loss.

**Figure 4:**
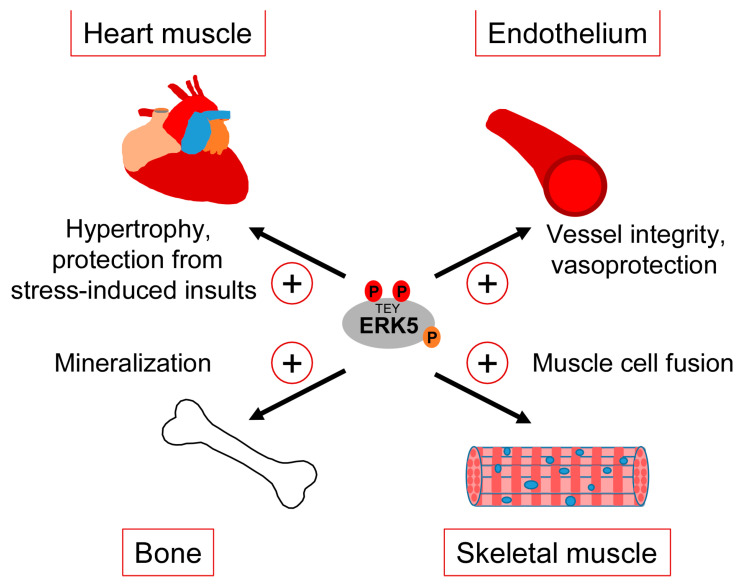
Tissue-sustaining effects of ERK5 in different mechanical stress-exposed tissues.

Clearly, our gaps in understanding the exact correlation between CDC42 and ERK5 signaling and the various controversies about the role of ERK5 in the mentioned studies warrant a more careful analysis of the interdependencies between CDC42 and ERK5 activity and their specific roles in cartilage and bone homeostasis. However, taking available genetic data and our observed ERK5 activation by bone-sustaining N-BPs into account, it seems fair to assume that ERK5 plays a protective rather than a destructive role in bone formation. Thus, similar to the endothelium, ERK5 activation in the bone might represent a physiological stress signal that ensures tissue integrity in response to sustained mechanical stimulation (Figure 4).

### 3.3. Heart and Skeletal Muscle

The consistently reported cardiac phenotype of the different original ERK5 knockout studies early on implied a role of the MEK5/ERK5 pathway in heart development [34]. However, the unapparent phenotype of the subsequently published cardiomyocyte-specific knockout mice suggested that the observed cardiac phenotype in the *Erk5*^−/−^ mice developed secondary to vascular dysfunction [33]. Nonetheless, several pieces of evidence still implicate the MEK5/ERK5 pathway in the regulation of heart muscle function, in particular in the context of stress responses such as the control of cardiac hypertrophy or the mediation of cardioprotection upon ischemic insults [97] (Figure 4). For instance, hypertrophic stimuli such as leukemia inhibitory factor (LIF) transiently activated ERK5 in cultured cardiomyocytes, and expression of a constitutively active mutant of MEK5β, a natural splice variant of MEK5 lacking most of its N-terminal PB1 domain, resulted in cardiomyocyte elongation as a consequence of serial sarcomere assembly [98]. Furthermore, cardiac-specific expression of an activated MEK5β transgene in mice induced eccentric hypertrophy, as indicated by the thinning and dilatation of the ventricular chamber without a cellular loss and mass reduction [98]. By contrast, transgenic cardiac expression of active MEK5α, a full-length splice variant of MEK5 with an intact PB1 domain, failed to induce cardiac hypertrophy but showed an increased recovery and cardioprotection after ischemia [99], suggesting that MEK5-dependent ERK5 activation may protect the heart from stress-induced insults (Figure 4). The discrepancy between both transgenic mouse models was proposed to result from a dominant-negative function of MEK5β [100]. However, a more recent study from Xin Wang’s group argues against that view, as conditionally cardiomyocyte-specific *Erk5* knockout mice, despite appearing phenotypically normal, exhibited decreased hypertrophic growth and fibrosis as well as increased cardiomyocyte apoptosis in response to the experimental induction of hypertrophic stress by transverse aortic constriction (TAC) [101]. This phenotype is reminiscent of that previously reported for *Mef2d*^−/−^ mice [102] and is in line with an enhanced hypertrophy seen in mice with cardiac-specific overexpression of *Mef2a* after TAC [103], further corroborating a role of the MEK5/ERK5/MEF2 signaling pathway in the hypertrophic stress response of the heart. A cardioprotective effect of the ERK5/MEF2 module is also supported by the observations that metabolic stress-induced cardiomyopathy was associated with decreased expression of ERK5, MEF2A, and MEF2D, and that the specific conditional deletion of *Erk5* in cardiomyocytes intensified this metabolic-induced cardiomyopathy [104]. These mice exhibited a decreased hypertrophic response and a lower capacity to withstand high fat diet-induced stress. They further showed an impaired cardiac contractility and increased cardiomyocyte apoptosis due to a mitochondrial dysfunction and an enhanced reactive oxygen species (ROS) production, which most likely resulted from the loss of proliferator-activated receptor γ co-activator 1α (Pgc-1α) expression that is essential for cardiac mitochondrial functions [104].

Comparably less information is available regarding the role of the ERK5 pathway in skeletal muscle, whose function likewise critically depends on mechanical cues [105]. It is well-known that factors of the MYOD and MEF2 family synergize to induce myoblast differentiation from fibroblasts, and that MEF2 family members play key roles in muscle development in *Drosophila* and in muscle regeneration in mammals [106]. It is therefore not surprising that MEK5/ERK5 signaling and its effectors KLF2 and KLF4 have also been implicated in muscle differentiation [107,108]. An early study reported that ERK5 was activated during differentiation of murine C2C12 myoblasts and that constitutive MEK5 activation was capable of inducing promoter activity of several myogenic genes in an ERK5-dependent manner [107]. Nishida’s group later suggested a specific role of an ERK5/KLF pathway in muscle cell fusion [108] (Figure 4). Interestingly, they observed that, in differentiating C2C12 myoblasts infected with a dominant-negative MEK5 or a control vector, ERK5-dependent gene expression critically relied on KLF2 and KLF4 but was apparently independent of MEF2 and MyoD [108]. Similar to ECs, both *KLF* genes were induced in a MEK5-dependent manner but specifically contributed to muscle cell fusion and not to MEF2/MYOD-dependent expression of muscle differentiation genes [108]. This suggests that during muscle differentiation, the ERK5/KLF pathway may operate independently of the activities of MyoD and MEF2 family transcription factors. It is therefore possible that the previously reported dependency of KLF2 and KLF4 expression on MEF2 [47,54] might not apply to all cell types and that ERK5, at least in some cellular contexts, may also control its effects independently via a separate ERK5/KLF module.

### 3.4. ERK5 in Cancer

Since the discovery and cloning of oncogenic RAS and RAF-expressing viruses in the late 1970s and early 1980s, MAPK pathways have attracted interest as promising targets for cancer therapy [109]. In various cancers, oncogenic driver mutations in the RAS/RAF/ERK1/2 MAPK pathway are common, leading to constitutive pathway activation, growth factor-independent proliferation, and the survival of the tumor cells [110]. The clear-cut tumor association of those mutations triggered the clinical development of several small molecule therapeutics inhibiting the ERK1/2 MAPK pathway (referred to as MAPKi in this review). These include inhibitors specific for oncogenic BRAF V600 (BRAFi) and compounds targeting its downstream kinase MEK1/2 (MEKi), which particularly proved successful as a combination therapy for advanced melanoma, where BRAF V600 mutations make up ~50–60% of all cases [111]. In contrast to the RAF/MEK/ERK pathway, activating mutations in components of the MEK5/ERK5 pathway are rare throughout the cancer entities and activation of the MEK5/ERK5 pathway in tumors is associated with overexpression of single components of the ERK5 pathway or deregulated receptor tyrosine kinase activity [112]. For a long time, the incentive to develop specific ERK5 inhibitors (ERK5i) for cancer therapy therefore remained relatively low. However, in the last decade, data have accumulated showing that the ERK5 pathway is frequently activated in various tumors and regulates several hallmarks of cancer, as already comprehensively covered in excellent reviews elsewhere [12,13,15]. Below, we will therefore limit our discussion on available literature implying the MEK5/ERK5 cascade as an important pathway that cooperates with RAS/RAF/MEK/ERK1/2 signaling in tumorigenesis. Moreover, we will summarize recent evidence on its emerging role as a compensatory pathway for ERK1/2, which sustains the proliferation and survival of various tumor cells under targeted MAPKi therapy.

The first clue for a potential crosstalk between ERK1/2 and ERK5 signaling in tumor cells dates back to the late 90s of the last century, when Melanie Cobb’s group reported the activation of ERK1/2 as well as of a C-terminally truncated kinase-proficient ERK5 mutant upon overexpression of oncogenic H-RAS V12 in HEK293 cells [28]. Surprisingly, oncogenic RAF-1 failed to activate this ERK5 deletion mutant, although RAF-1 was still required for RAS V12-mediated ERK5 activation [113]. RAS V12-mediated ERK5 activation was also confirmed by an independent study by Nishida’s group, but in this case was found to be cell type-specific [114]. Consistently, Kato et al. failed to observe RAS V12-induced ERK5 activation in HeLa cells and did not observe a requirement of RAS for EGF-induced ERK5 activation [7]. Hence, oncogenic RAS is apparently not always associated with ERK5 activation. This view is consistent with our own findings that human NRAS-mutant melanoma cell lines and patient biopsies frequently but not generally show increased ERK5 activity [37]. In agreement, several other studies excluded a general crosstalk between ERK5 and ERK1/2 signaling, since expression of an active MEK1 mutant failed to activate ERK5 and a constitutive active MEK5D was unable to activate MAPKs other than ERK5 in various cells [6,50,115]. Still, oncogenic RAF-1 as well as constitutively active MEK1 were found to cooperate with constitutively active MEK5D in the transformation of NIH3T3 cells [113,114]. This has been attributed to a synergistic activation of nuclear factor kappa-light-chain-enhancer of activated B cells (NF-κB) by the ERK5-dependent activation of p90 ribosomal S6 kinase (p90RSK) [116]. ERK5 was also required for SRC-mediated transformation and cytoskeletal disruption in NIH3T3 cells [115]. Neither the inhibition of ERK1/2 nor ERK5 alone was sufficient to restore the disturbed actin cytoskeleton in SRC-transformed cells, suggesting an independent cooperation of both pathways in the SRC-induced transformation [115]. Strikingly, the authors further observed that MEK inhibition augmented ERK5 activity in SRC-transformed cells, as judged by an increased nuclear localization of ERK5 and an enhanced MEF2-luc reporter activity under these conditions [115]. This provided the first evidence that ERK5 activation might serve as an escape route in order to compensate for MAPKi, allowing tumor cells with an activated RAS/RAF/MEK/ERK pathway to resist the targeted MAPKi therapy.

**Figure 5:**
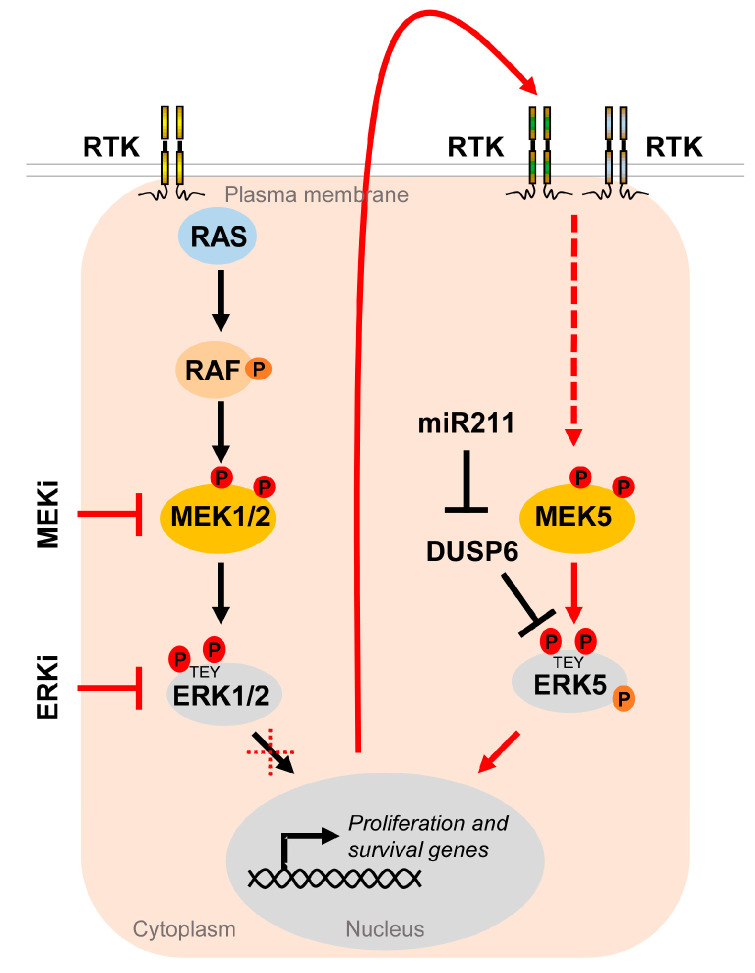
The MEK5/ERK5 pathway serves as an escape route to promote proliferation and survival of cancer cells under MAPKi. Oncogenic driver mutations in components of the RTK/RAS/RAF/MEK/ERK1/2 pathway lead to hyperactivation of the MEK/ERK1/2 cascade in multiple cancers. Existing inhibitors of the ERK1/2 pathway (MAPKi) targeting MEK1/2 (MEKi) or ERK1/2 (ERKi) trigger compensatory activation of the MEK5/ERK5 pathway via stimulation of different receptor tyrosine kinases (RTK) [37,119,120] in order to allow tumor cells to escape MAPKi-induced cell cycle arrest and apoptosis. Additionally, ERK5 activity appears to be upregulated by DUSP6 regulation, an ERK5 specific dual specificity phosphatase, whose inhibition by miR211 was shown to increase basal ERK5 phosphorylation [121].

One tumor cell type, in which this concept has been extensively tested in the last years, is malignant melanoma. An overwhelming majority of ~80% of skin cutaneous melanomas share an activating driver mutation in either the *BRAF* or *NRAS* oncogene, leading to hyperactivation of the MEK-ERK1/2 cascade [111]. Existing therapeutics targeting MEK1/2 and upstream kinases such as BRAF are prone to develop extrinsic resistance [117]. Moreover, NRAS-mutant melanoma, which constitutes ~25% of all melanoma cases, do not significantly benefit from those therapies, as they commonly lack oncogenic BRAF mutations and show a high degree of intrinsic resistance to MEKi [111,118]. An increased ERK5 signaling conferring MAPKi insensitivity in part mediates these resistance mechanisms. In our previous publication, we have shown that both BRAF- and NRAS-mutant melanoma cell lines significantly upregulated ERK5 signaling when subjected to MEKi using trametinib, selumetinib, binimetinib or cobimatinib, or ERK1/2 inhibition (ERKi) using GDC-0994 [37] (Figure 5).

The compensatory ERK5 activation occurred in a delayed fashion and was sensitive to a platelet-derived growth factor receptor (PDGFR) [37], whose upregulation, among that of other receptor tyrosine kinases, frequently contributes to MAPKi resistance in melanoma [122]. Using the ERKi SCH772984, Benito-Jardon and colleagues alternatively described the IGF1R-mediated activation of the MEK5/ERK5 cascade as an escape route used by melanoma cells in order to circumvent ERKi and promote cell proliferation [119]. Another report has shown that intrinsic resistance to combined BRAFi and MEKi can efficiently be curbed by co-inhibiting ERK5, either by the pharmacological ERK5i XMD8-92 or by expressing an shRNA against ERK5 [123]. We have recently reported corroborating results on a xenograft model using NRAS-mutant melanoma cells: the co-inhibition of the MAPK/ERK pathway with trametinib and the MEK5/ERK5 cascade using the ERK5i XMD8-92 was effective in preventing melanoma expansion [37]. Tusa et al. similarly observed melanoma suppression by the treatment of BRAF-mutant human xenografts with a combination of the BRAFi vemurafenib and the ERK5i XMD8-92 [124]. Conversely, Lee et al. recently reported that expression of miR211, a micro RNA suppressing expression of the ERK5-specific dual specificity phosphatase *DUSP6*, which removes phosphorylation at the MEK5-targeted TEY motif of ERK5, led to an increased ERK5 basal phosphorylation, and thereby augmented the tumor growth of *BRAF* mutant melanoma cells in vivo and their resistance to the BRAFi vemurafenib in vitro [121]. These data show that efficient repression of the ERK5, along with the MEK/ERK1/2 or BRAF inhibition, could be a more effective treatment of melanoma that may lower the rate of tumor resistance.

ERK5 dependencies of tumors exposed to MAPKi are not limited to melanoma (Table 2).

In both wild-type and KRAS-mutant colon cancer cells, treatment with the pharmacological MEKi PD0325901 resulted in a marked upregulation of ERK5 phosphorylation [126]. Co-treatment of those cells with the ERK5i XMD8-92 significantly improved the tumor-suppressive effect of PD0325901 [126]. Vaseva et al. have similarly shown that the suppression of ERK1/2 in KRAS-mutant pancreatic ductal adenocarcinoma (PDAC) induced ERK5 activation and subsequently stabilized cMYC. The additional inhibition of ERK5, however, hampered the ERK5-mediated cMYC stabilization and revealed a synergistic action in preventing PDAC proliferation [120]. In KRAS-mutant non-small cell lung cancer (NSLC), a CRISPR/Cas9 screen revealed *ERK5* loss as a sensitizer for the MEKi cobimetinib [125]. Similar to other tumor entities, MEK inhibition by cobimetinib increased ERK5 activation in at least a fraction of NSCLC cell lines, and a combination of cobimetinib and ERK5 shRNA led to a decreased tumor volume in xenotransplantation experiments [125]. In triple negative breast cancer (TNBC) cells, Hoang et al. showed that the inhibition of MEK1/2 and MEK5 using a pan-MEKi drastically decreased the migration potential of the cells compared to MEK1/2 or MEK5 inhibition alone. Even though the authors failed to show the comparative differences in vivo, the employed pan-MEKi SC-151 was effective in reducing cell viability, cellular migration, and invasion in a sub-group of aggressive TNBC cell lines [127].

Together, these results suggest that ERK5 signaling may constitute a conserved intrinsic resistance mechanism when tumors are targeted with MAPKi. This implies that combined MAPKi/ERK5i may efficiently suppress the frequently observed resistance of various tumors with a deregulated RAS/RAF/MEK/ERK pathway.

One issue emerging from those data, as well as from the discovery of a potentially kinase-independent transcriptional function of ERK5, is whether tumor-suppressive strategies should merely target the kinase activity or additionally aim to suppress the ERK5 transcriptional capacity. Besides XMD8-92, several other small molecule inhibitors for ERK5 have been developed (reviewed in [128]). Surprisingly, recent data with novel highly specific ERK5 kinase inhibitors suggest that efficient ERK5 kinase inhibition may induce paradoxical transcriptional activation of ERK5 [129]. Whether this may reflect an unusual peculiarity of the investigated compounds remains to be investigated. However, it seems as if slightly less specific dual inhibitors blocking both transcriptional activity and ERK5 kinase function, such as XMD8-92 [31] or inhibitors targeting ERK5 localization, such as the CDK/ERK5 multi kinase inhibitor TG02, which proved useful for the treatment of myeloma [130], might be a better choice.

## 4. Manipulating ERK5—A Double-Edged Sword

Besides its role as a classical growth factor-activated MAPK pathway, the MEK5/ERK5 cascade importantly modulates mechanical stress responses throughout the body in different tissues, including vascular endothelium, bone, cartilage, and muscle. Over the years, several knockout studies in mice as well as studies with human primary ECs have established the MEK5/ERK5 pathway as a key player, maintaining tissue homeostasis in the cardiovascular system. These studies revealed a critical requirement of ERK5 for survival and the mediation of shear stress signaling in ECs, thus imparting an overall protective effect on the vascular endothelium. In bone and cartilage, ERK5 clearly facilitates bone formation by enhancing anabolic processes and suppressing catabolic activities. Given that statins and N-BPs, drugs used for the treatment of hyperlipidemia or osteoporosis, respectively, can induce ERK5 activity, it is safe to presume that ERK5 upregulation is beneficial for patients with such diseases. However, in light of the emerging role of the MEK5/ERK5 pathway as a tumor-promoting pathway, efforts to increase the ERK5 activity might also be associated with an increased risk of cancer. On the other hand, the co-inhibition of ERK5, along with MAPKi, could be a great leap forwards in treating therapy-resilient cancers such as melanoma. Similarly, as ERK5 and its downstream targets appear to be malicious in CCM, the suppression of the ERK5 pathway could be fruitful. Considering its indispensable role for cardiovascular health and endothelial integrity, targeting ERK5 in such diseases is definitively a double-edged sword, as it may foster cardiovascular disease or life-threatening hemorrhages. It should be noted, however, that in none of the published intervention studies in mice using the pharmacological ERK5 inhibitor XMD8-92 have apparent cardiovascular defects been observed [31,37,120,123,124], raising hope that ERK5 may safely be targeted. It is unclear why XMD8-92 treatment failed to induce similar cardiovascular defects as in the knockout studies. However, it is unlikely that this can be accounted for by mere interference with the kinase function by XMD8-92, as it was also able to suppress the transcriptional activation capacity of ERK5 in luciferase assays [31]. Certainly, more work is required in order to resolve this issue and to elucidate how exactly the catalytic and transcriptional activities of ERK5 cooperate to regulate its diverse functions. Only a complete understanding of the distinct ERK5 activities and the consequences arising from the specific intervention with these functions will enable us to fully exploit the potential of ERK5-directed therapies. Considering the strikingly diverse roles of ERK5, future studies at any rate should involve a rigorous multi-disciplinary scientific discourse before ERK5 manipulation strategies are transferred into clinical settings.

## Figures and Tables

**Table 1:** List of published functionally relevant ERK5 phosphorylation sites confirmed by kinase assay or mutagenesis.

Human ERK5 Peptide Position(s) ^1^ (Reference Peptide Sequence Uniprot Q13164)	Mouse Equivalent Position(s) (Reference Peptide Sequence Uniprot Q9WVS8)	Domain	Proposed Kinase(s)	Reference
T219 and Y221	T219 and Y221	KD ^2^	MEK5	[6]
T733, S770, S774, and S776	T723, S760, S764, and S766	TAD	ERK5 (auto)	[20]
T733, S770, S774, and S776	T723, S760, S764, and S766	TAD	ERK5 (auto) ^3^	[23]
T733	T723	TAD	CDK5	[24]
S707, T733, S754, and S774	S697, T723, S744, and S764	TAD	CDK1 ^4^	[25]
S567, S720, S731, T733, and S803	S567, S710, S721, T723, and S793	TAD	CDK1 ^4^	[26]
T733	T733	TAD	ERK2 ^4^	[23]

^1^ Labelling of the provided sites is based on the reference amino acid sequences listed in the Uniprot database (available at: https://www.uniprot.org/uniprot/Q13164 (accessed on 7 July 2021) and https://www.uniprot.org/uniprot/Q9WVS8 (accessed on 7 July 2021)). Positions may slightly vary from those in the original reports, in which labeling was frequently based on the position in the utilized flag-ERK5 construct originally made by Kato et al. [6], which lacks the start codon; ^2^ KD: kinase domain (aa 76–406), TAD: transactivation domain (aa 664–789) [21]; ^3^ phosphorylation sites were not individually determined by kinases assay but assessed by Western blots using a multisite-specific antibody raised against C-terminally phosphorylated ERK5; ^4^ responsible kinase concluded based on sensitivity to an employed pharmacological inhibitor without demonstration of direct phosphorylation by kinase assay.

**Table 2:** List of studies showing improved tumor suppression upon combined MAPKi and ERK5 pathway inhibition in vivo.

Tumor Type	Employed Inhibitor Combinations In Vivo		Reference
	MAPKi	ERK5i	
BRAF mutant melanoma	Vemurafenib (BRAFi) + Trametinib (MEKi)	XMD8-92	[123]
BRAF mutant melanoma	Vemurafenib (BRAFi)	XMD8-92	[124]
NRAS mutant melanoma	Trametinib (MEKi)	XMD8-92	[37]
KRAS mutant non-small cell lung cancer	Cobimetinib (MEKi)	shERK5	[125]
KRAS mutant pancreatic ductal adenocarcinoma	SCH772984 (ERKi)	XMD8-92	[120]
